# DHA Modulates *Pparγ* Gene Expression Depending on the Maturation Stage of 3T3-L1 Adipocytes at Time of Exposure

**DOI:** 10.3390/ijms262311514

**Published:** 2025-11-27

**Authors:** Natalia Grigorova, Zhenya Ivanova, Tanya Tacheva, Ekaterina Vachkova, Ivan Penchev Georgiev

**Affiliations:** 1 Department of Pharmacology, Animal Physiology, Biochemistry and Chemistry, Faculty of Veterinary Medicine, Trakia University, 6000 Stara Zagora, Bulgaria; zhenya.ivanova.12@trakia-uni.bg (Z.I.); ekaterina.vachkova@trakia-uni.bg (E.V.); ivan.georgiev@trakia-uni.bg (I.P.G.); 2 Department of Medicinal Chemistry and Biochemistry, Faculty of Medicine, Trakia University, 6000 Stara Zagora, Bulgaria; tanya.tacheva@trakia-uni.bg

**Keywords:** docosahexaenoic acid (DHA), *Pparγ*, *Gpr120*, adiponectin, omega-3 fatty acids, adipocyte maturation stage, adipogenesis, lipolysis

## Abstract

Omega-3 fatty acids, particularly DHA, are potent modulators of adipose tissue biology. However, reported effects on adipogenesis vary with dose and adipocyte maturation. We examine the effects of prolonged exposure to 60 μM DHA on lipogenesis, lipolysis, and glucose uptake in 3T3-L1 adipocytes. DHA was administered either during early differentiation (days 1–9, followed by maturation in maintenance medium) or during the mature stage (days 9–18), with all analyses performed on day 18. DHA supplementation of immature adipocytes markedly inhibited adipogenesis. Intracellular lipid accumulation was reduced by 56%, accompanied by a strong downregulation of *Pparγ* and *Fasn*, and undetectable levels of *Gpr120*. Correspondingly, *Slc2a4* (GLUT4) was suppressed, accompanied by a 44% reduction in glucose uptake. The strong suppression of the adipogenic program and increased *Cpt1*-linked mitochondrial β-oxidation in immature adipocytes align with DHA’s well-known anti-inflammatory and ROS-lowering effects. When applied to mature adipocytes at the same dose and duration, DHA also decreased intracellular lipid accumulation and glucose utilization, although more modestly (by 30% and 8%, respectively). However, unlike in immature adipocytes, the lipolysis rate in mature cells was increased by 34% and *Pparγ* expression remained unchanged, indicating an entirely different metabolic pathway of modulation. In mature adipocytes, DHA promoted lipid mobilization rather than the general suppression of lipogenesis and glucose uptake. Overall, these findings highlight a distinct, stage-specific antiadipogenic mechanism of DHA action, but also underline that its context-dependent effects may become detrimental when high physiological doses overlap with conditions of energy surplus.

## 1. Introduction

Omega-3 polyunsaturated fatty acids (omega-3; PUFAs) have a profound impact on adipocyte biology and are renowned for their positive effects on the health status of adipose tissue and the overall well-being of the organism [1,2,3]. They are well-known as strong anti-inflammatory agents, which benefit various conditions, including metabolic syndrome, insulin resistance, rheumatoid arthritis, inflammatory bowel and cardiovascular diseases [4,5,6,7,8]. Nonetheless, studies exploring the influence of PUFAs on adipogenesis and their modulation of adipocytes’ “buffer capacity” yield notably contradictory findings. Animal and human investigations could be generally divided into two main categories: studies that demonstrate pro-adipogenic effects of PUFAs [9,10,11,12,13,14] and studies that reveal contrasting anti-adipogenic impact [15,16,17,18,19].

Omega-3 fatty acids and their intracellular-derived metabolites interact with crucial adipocyte transcriptional factors and membrane receptors, further modulating intrinsic metabolic pathways involved in adipogenic program [4,11,14,15,18]. Recognized as natural ligands for peroxisome proliferator-activated receptor gamma (PPARγ) and G-protein-coupled receptor 120 (GPR120), PUFAs predominantly promote insulin sensitivity, thus glucose and free fatty acid influx, intracellular triglyceride deposition, and health expansion in adipocytes. These PPARγ-, GPR120-provoked benefits are often associated with an upregulation of adiponectin—a pivotal adipocine that maintains the health status of white adipose tissue, thereby mitigating the systemic energy balance [12,13,20,21,22]. In addition, PUFAs are shown to inhibit lipolysis in mature adipocytes by reducing the expression of adipose triglyceride lipase (ATGL) and hormone-sensitive lipase (HSL) [13,23,24], further favoring adipocyte expansion and thus mitigating the risk of ectopic lipid deposition [4,25,26].

Numerous studies underline a precisely contrasting impact. They report several mechanisms by which PUFAs exert anti-adipogenic and weight-lowering effects. Most of these studies, along with suppression of pro-adipogenic genes, point out enhanced mitochondrial lipid oxidation and ameliorated adipocine profile of secretion [2,15,17,18,19,27]. Notably, a lot of them established increased lipolysis rate and even adipocyte apoptosis, which is interpreted as a positive outcome in the weight-lowering aspect, but in fact it poses a health risk due to the risk of lipid overload in the circulation [17,28,29,30]. These increased free fatty acids could lead to various adverse consequences, including insulin resistance, hepatic steatosis, and β-cell dysfunction [31,32,33,34].

Increasing research suggests that each omega-3 fatty acid has a distinct mechanism of action, and it has become evident that considering them collectively or recommending them as a single group is no longer sufficiently justified [35,36]. The enormous diversity in experimental approaches makes it difficult to derive specific, universally applicable recommendations even for a single type of omega-3 fatty acid. Variability in results arises from numerous factors such as dosing, fat content, and diet type [37,38,39]. Even focusing on the effects of docosahexaenoic acid (DHA), referred to as the most biologically active omega-3, with a pronounced antiadipogenic effect and emerging as a promising antitumor biomolecule [4,15,18,40,41], we fail to underline a unified trend of action and recommendation of use.

Therefore, the current study aimed to compare the impact of the same dose and period of application of DHA in immature and already mature 3T3-L1 adipocytes in vitro, focusing on their intracellular lipid deposition, lipolysis, glucose uptake, and gene expression of crucial pro-adipogenic, mitochondrial, oxidation, and lipolysis-associated factors.

## 2. Results

### 2.1. Pre-Experimental Procedure: Evaluation of 0.05% (v/v) Ethanol Effects on Adipogenesis

**Figure 1:**
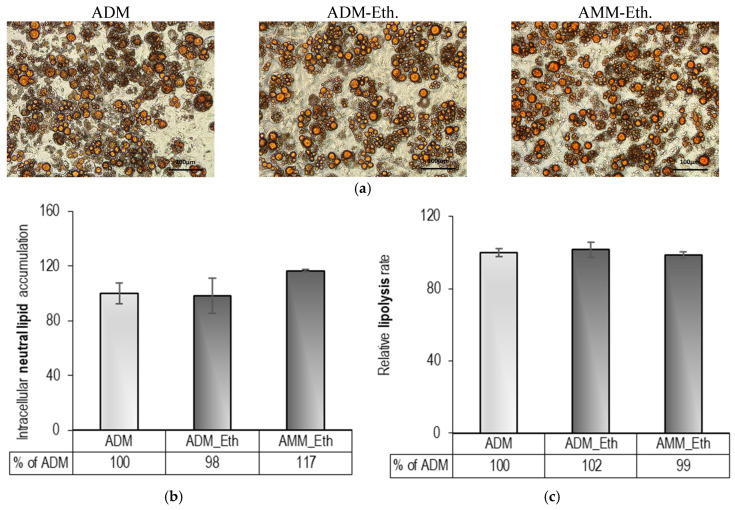
Impact of 0.05% (*v*/*v*) ethanol on 3T3-L1 adipocytes’ lipid accumulation and lipolysis: (**a**) Oil Red O-stained cell images were captured at 20× magnification (bars = 100 µm), (**b**) spectrophotometric analysis of intracellular neutral lipid accumulation, and (**c**) lipolysis rates in differentiating and mature cells. Abbreviations: ADM—untreated control; ADM-Eth—treated with 0.05% (*v*/*v*) ethanol during 9-day differentiation; AMM-Eth—treated from day 10 to 18 in mature cells. Data are shown as mean ± SEM (*n* = 6), expressed as a percentage of ADM. No statistically significant differences were observed.

Studies have demonstrated that ethanol, included in adipocyte culture medium at a concentration lower than 0.1% (*v*/*v*), does not exert a detrimental effect on the viability of 3T3-L1 preadipocytes [28,42]. Consistent with these findings, our previous experiments verified that DHA dissolved in 0.1% (*v*/*v*) ethanol and applied to the culture media for nine days on differentiating adipocytes did not exert any cytotoxicity [43]. However, it remains questionable whether the ethanol used as a diluent of DHA in the in vitro testing influences intracellular lipid accumulation and the adipocyte lipolysis rate, which could compromise the obtained results. Therefore, pre-experimental testing with 0.05% (*v*/*v*) ethanol was assessed before the experiment commenced. The ethanol was administered for nine days to both differentiating (immature) and already differentiated (mature) 3T3-L1 adipocytes. The data presented in Figure 1 indicated no reduction in intracellular lipid deposition or increased lipolysis in the treated cells. Consequently, we assumed that the inclusion of 0.05% (*v*/*v*) ethanol in the culture media does not provoke a non-specific effect on adipogenesis, and the chosen concentration was suitable for the experiment goal.

### 2.2. DHA Influence on the Viability of Immature and Mature 3T3-L1 Adipocytes

**Figure 2:**
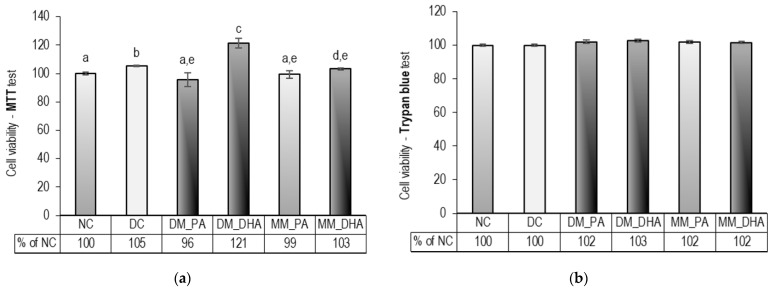
DHA impact on 3T3-L1 adipocyte viability via (**a**) MTT and (**b**) Trypan blue assays on day 18. Abbreviations: NC—non-differentiated control; DC—differentiated control; DM_PA—differentiating cells treated with 60 µM PA (days 1–9); DM_DHA—differentiating cells treated with 60 µM DHA (days 1–9); MM_PA—mature cells treated with 60 µM PA (days 10–18); MM_DHA—mature cells treated with 60 µM DHA (days 10–18). Results (mean ± SEM) are presented as per cent relative to DC (*n* = 6). Different letters indicate statistically significant differences between groups at *p* < 0.05.

The MTT and Trypan blue exclusion assays were performed at the end of the experimental period. The results, presented in Figure 2, showed no cytotoxic effect and even an increase in mitochondrial activity (MTT) in DM_DHA (*p* < 0.01) and MM_DHA (*p* < 0.05) compared to DC.

### 2.3. DHA Impact on Lipid Accumulation in Immature and Mature 3T3-L1 Adipocytes

**Figure 3:**
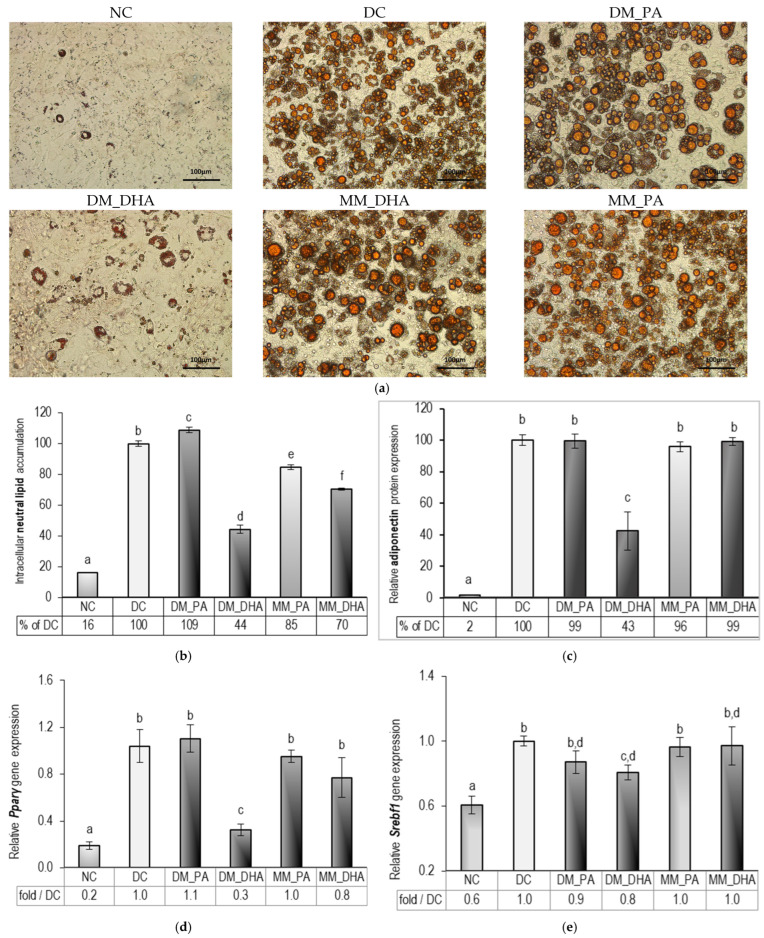

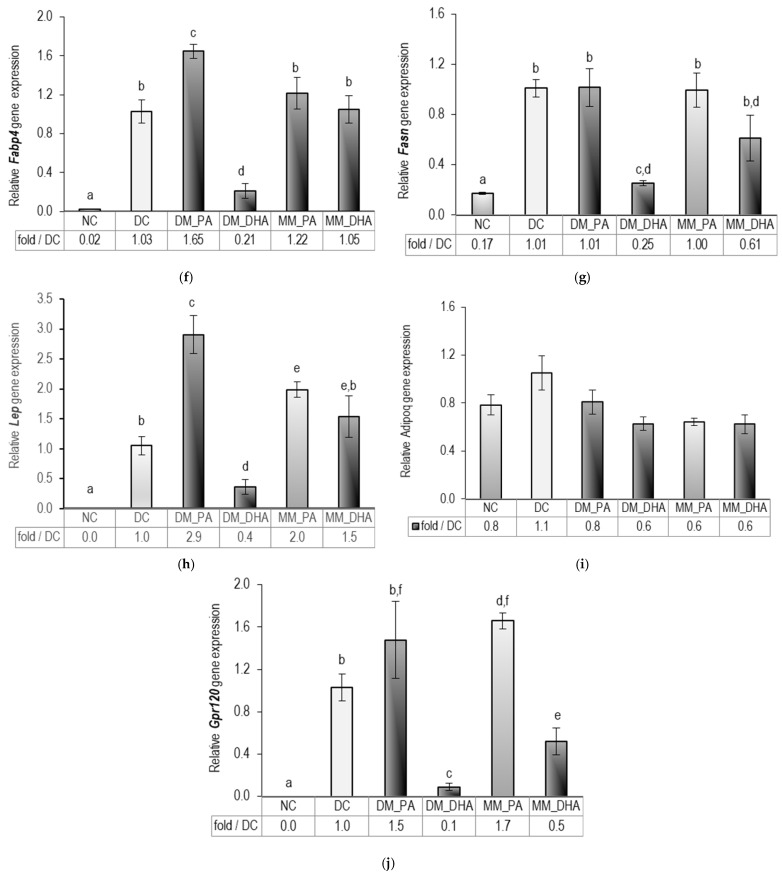
DHA impact on 3T3_L1 adipogenesis: (**a**) Microscopic images of Oil Red O stained intracellular lipid droplets in 3T3-L1 were captured at 20× magnification (bars = 100 µm), (**b**) spectrophotometric analysis of intracellular neutral lipid accumulation, (**c**) adiponectin protein expression; and relative mRNA gene expression of (**d**) peroxisome proliferator-activated receptor gamma (*Pparγ*), (**e**) Sterol regulatory element binding transcription factor 1 (*Srebf1*), (**f**) fatty acid binding protein 4 (*Fabp4*), (**g**) fatty acid synthase (*Fasn*), (**h**) leptin receptor (*Lep*), (**i**) adiponectin (*Adipoq*), and (**j**) G protein-coupled receptor 120 (*Gpr120*). Abbreviations: NC—non-differentiated control; DC—differentiated control; DM_PA—differentiating cells treated with 60 µM PA (days 1–9); DM_DHA—differentiating cells treated with 60 µM DHA (days 1–9); MM_PA—mature cells treated with 60 µM PA (days 10–18); MM_DHA—mature cells treated with 60 µM DHA (days 10–18). Results (mean ± SEM) (*n* = 5) are presented relative to DC. Different letters indicate statistically significant differences between groups at *p* < 0.05.

Figure 3a shows Oil Red O-stained microscopic images at the end of the experiment. Pronounced adipogenesis is evident in all images from the differentiated groups, while only occasional spontaneous adipogenesis was established in NC. Treatment of 3T3-L1 cells with 60 µM DHA during adipogenesis (days 1–9) markedly inhibited preadipocyte differentiation. Microscopic imaging reveals that in the DM_DHA group, the monolayer remained intact. Most cells exhibited only small lipid droplets resembling the sporadic induction seen in NC. Only single cells underwent clear adipogenic differentiation, characterized by a fine-grained structure of the lipid inclusions. In contrast, no apparent anti-adipogenic effect of DHA was detected in mature adipocytes by microscopic assessment.

To validate the findings from the microscopic images in Figure 3a, we further quantified the neutral lipid content in the Oil Red O-stained cell cultures using spectrophotometric analysis of their isopropanol extracts. Figure 3b presents the optical densities as a percentage relative to the DC after exclusion of the spontaneous adipogenesis measured in the NC. The results align closely with the visual changes seen under the microscope. We observed an approximately 80% difference in lipid accumulation between the NC and DC groups (*p* < 0.01)—a logical consequence of the induced adipogenesis. DHA supplementation, however, led to a substantial decrease in lipid deposition compared to the DC group (*p* < 0.01), with a more pronounced suppression in DM-DHA (by 54%) than in MM-DHA (by 30%), clearly underlying the anti-adipogenic effect of DHA. Further, we investigated the expression levels of critical early and late adipogenic markers. The results are presented in Figure 3d–j. Consistent with these findings, peroxisome proliferator-activated receptor gamma (*Pparγ*), sterol regulatory element binding transcription factor 1 (*Srebf1*), fatty acid-binding protein 4 (*Fabp4*), fatty acid synthase (*Fasn*), leptin receptor (*Lep*) mRNA levels and G protein-coupled receptor 120 (*Gpr120*), were significantly reduced in the DM_DHA group compared to DC (*p* < 0.01), while in MM_DHA, only *Gpr120* expression was notable downregulated (*p* < 0.05). Gene expression profiles in DM_PA and MM_PA groups—used as isoenergetic controls for DHA—remained unchanged or modestly increased relative to DC, further supporting the specific role of DHA in mediating antiadipogenic gene expression effects.

Adiponectin protein expression was quantified by the sandwich ELISA method. Results are presented in Figure 3c as a percentage relative to the DC. DHA supplementation significantly reduced adiponectin concentration in the cell supernatants by 57%, whereas no significant differences were observed in MM_DHA compared to DC.

### 2.4. DHA Impact on Glucose Uptake in Immature and Mature 3T3-L1 Adipocytes

**Figure 4:**
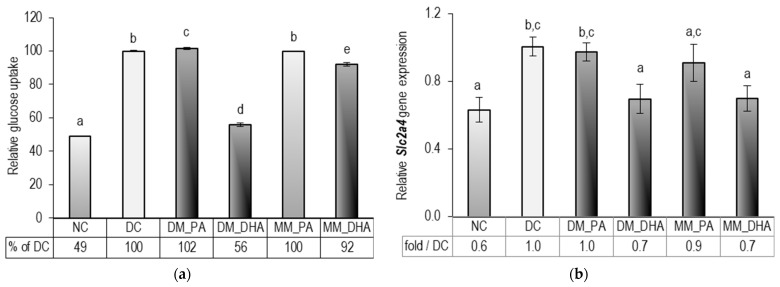
Glucose uptake (**a**) and *Slc2a4* gene expression (**b**) in 3T3-L1 cells. Abbreviations: NC—non-differentiated control; DC—differentiated control; DM_PA—differentiating cells treated with 60 µM PA (days 1–9); DM_DHA—differentiating cells treated with 60 µM DHA (days 1–9); MM_PA—mature cells treated with 60 µM PA (days 10–18); MM_DHA—mature cells treated with 60 µM DHA (days 10–18). Results (mean ± SEM) (*n* = 5) are presented relative to DC. Different letters indicate statistically significant differences between groups at *p* < 0.05.

To assess glucose uptake by adipocytes at the end of the experiment, we determined the difference between the initial glucose concentration in the culture medium and the remaining concentration in each cell supernatant after 48 h. Figure 4 displays these values as a percentage of the differentiated control (DC), adjusted for the baseline glucose uptake measured in the NC. We evaluated a significant reduction in glucose uptake in DM-DHA group (*p* < 0.01) with a marked downregulation of solute carrier family 2 member 4 (Slc2a4) gene expression. A less pronounced, yet statistically significant decrease was also observed in the MM-DHA group (*p* < 0.01).

### 2.5. DHA Impact on the Lipolysis in Immature and Mature 3T3-L1 Adipocytes

**Figure 5:**
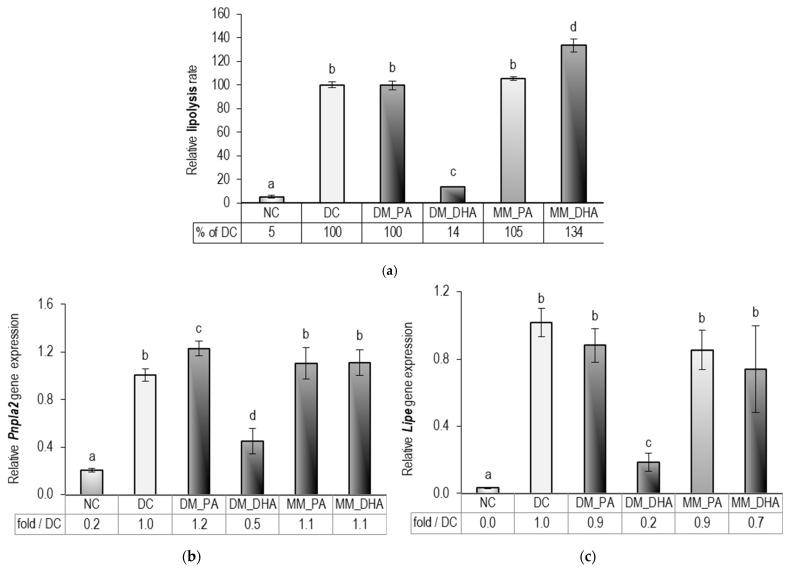
DHA impact on the lipolysis in immature and mature 3T3-L1 adipocytes: (**a**) relative lipolysis rate as a percentage of DC, (**b**) fold-change mRNA expression of patatin-like phospholipase domain containing 2 (*Pnpla2*), and (**c**) lipase, hormone-sensitive (*Lipe*) relative to DC. Abbreviations: NC—non-differentiated control; DC—differentiated control; DM_PA—differentiating cells treated with 60 µM PA (days 1–9); DM_DHA—differentiating cells treated with 60 µM DHA (days 1–9); MM_PA—mature cells treated with 60 µM PA (days 10–18); MM_DHA—mature cells treated with 60 µM DHA (days 10–18). Results (mean ± SEM) (*n* = 5) are presented relative to DC. Different letters indicate statistically significant differences between groups at *p* < 0.05.

In this study, we analyzed adipocyte lipolysis and the expression of key lipolysis-related genes on day 18 (Figure 5). Lipolysis was assessed by measuring glycerol levels in the cell supernatants. Because adipocytes have low glycerol kinase activity and do not reabsorb glycerol—unlike free fatty acids [44]—glycerol concentration serves as a reliable indicator of lipolytic activity [45]. The lipolysis rate in DM-DHA was only 9% higher than in NC (*p* < 0.01) but more than 85% lower than in DC and MM-DHA (*p* < 0.01), highlighting the dependence of the DHA effect on adipocyte maturity. In contrast, comparison between MM_DHA and DC revealed a statistically significant 34% increase in lipolysis following DHA supplementation in mature adipocytes (*p* < 0.01) (Figure 5a).

Gene expression of patatin-like phospholipase domain-containing 2 (*Pnpla2*), encoding ATGL, and hormone-sensitive lipase (*Lipe*), encoding HSL, was significantly downregulated in the DM_DHA group compared to both DC and MM_DHA (*p* < 0.01), as shown in Figure 5b,c. This reduction was consistent with the markedly suppressed lipolytic activity observed in the DM-DHA group. In contrast, the 34% increase in lipolysis in the MM-DHA group was not accompanied by a corresponding upregulation of either gene. This discrepancy between lipolysis rate and mRNA levels suggests that in mature adipocytes, lipolytic regulation is primarily driven at the protein or post-translational level, underscoring the distinct mechanisms governing DHA responses in immature versus mature cells.

### 2.6. DHA Impact on Carnitine palmitoyltransferase 1 (Cpt1) Gene Expression in Immature and Mature 3T3-L1 Adipocytes

**Figure 6:**
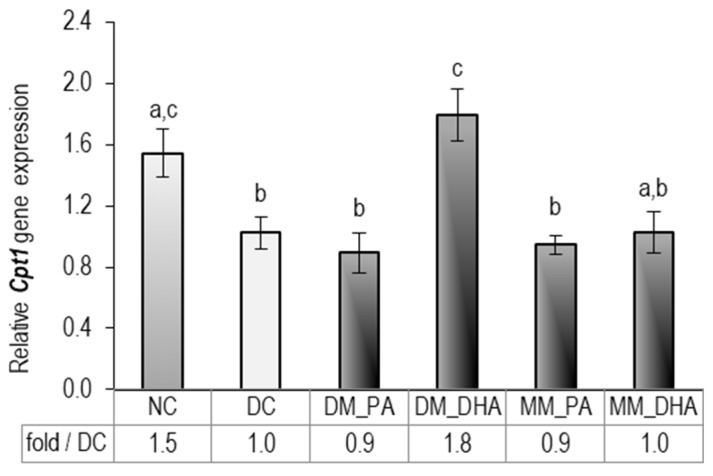
DHA impact on *Cpt1* gene expression in immature and mature 3T3-L1 adipocytes. Abbreviations: NC—non-differentiated control; DC—differentiated control; DM_PA—differentiating cells treated with 60 µM PA (days 1–9); DM_DHA—differentiating cells treated with 60 µM DHA (days 1–9); MM_PA—mature cells treated with 60 µM PA (days 10–18); MM_DHA—mature cells treated with 60 µM DHA (days 10–18). Results (mean ± SEM) (*n* = 5) are presented relative to DC. Different letters indicate statistically significant differences between groups at *p* < 0.05.

At the end of the experiment, we quantified *Cpt1* mRNA across groups relative to DC, as an indicator for β-oxidation capacity. It is well established that CPT1 protein is the rate-limiting gatekeeper of fatty-acid oxidation, converting long-chain acyl-CoA to acylcarnitine for mitochondrial import [46]. As shown in Figure 6, *Cpt1* was significantly downregulated in DC, DM-PA, and MM-PA compared to NC. Concerning DHA treatment, *Cpt1* gene expression was almost 2-fold upregulated in DM_DHA related to DC, while in MM-DHA it remained unchanged, suggesting that enhanced β-oxidation most likely accounts for the lipid-reducing effects observed in immature, rather than mature adipocytes treated with DHA.

## 3. Discussion

This study highlights the significance of adipocyte maturity in regulating cellular carbohydrate and lipid metabolism following DHA supplementation. To evaluate, in a comparative aspect, the preventive and therapeutic effects of DHA on obesity, we utilized 3T3-L1 cells, which are widely acknowledged as the most appropriate cell line for such studies [47]. These preadipocytes were exposed to DHA during and after adipogenic induction over a period of nine days, and the effect was reviewed in the context of three control groups: negative, positive, and isoenergetic—PA supplemented.

Preadipocytes exposed to 60 µM DHA from the onset of adipogenic induction exhibited negligible differentiation, evidenced by a significant reduction in intracellular neutral lipid accumulation. At the end of the experiment, we observed predominantly undifferentiated cells under microscopic investigation despite an excess of nutrient supply and the application of specific adipogenic inducers. The lipolysis rate and glucose uptake in this group were also markedly inhibited, while cell viability was preserved and even slightly increased. Previously, we observed a similar effect: when DHA was supplemented during eight days of adipogenesis in 3T3-L1 cells, it inhibited the expression of the *Pparγ* gene, which in turn blocked the cascade of mechanisms leading to intracellular lipid accumulation and glucose uptake in the treated cells [43]. The observed result was based entirely on the precise intracellular modulation of adipogenesis rather than the frequently cited reduction in cell viability [8,17,40]. In the current study, 3T3-L1 preadipocytes were exposed to DHA supplementation for nine days, followed by a subsequent nine-day culture in AMM (DMEM with high glucose and insulin). The findings from these experiments suggest that the application of DHA during differentiation interferes with adipogenic regulatory mechanisms, which are disrupted, thereby limiting the initial and further adipogenic potential of 3T3-L1 cells, regardless of the nutrient-rich environment.

*Pparγ* is a transcription factor that triggers intracellular lipid accumulation within adipocytes. The upregulation of *Pparγ* is indispensable for adipogenesis, and to date, no other transcription factor has been identified that can compensate for its absence [48,49,50,51,52]. It “orchestrates” a comprehensive network of transcriptional changes during adipocyte differentiation, causing preadipocytes to exit the cell cycle of proliferation and commence the expression of adipocyte-specific genes, such as *Gpr120*, *Fabp4*, *Fasn*, *Slc2a4*, *Adipoq*, and *Lep*. Although these genes represent only a fraction of those modulated by *Pparγ*, they are central players in the regulation of whole-body energy balance and are highly indicative of adipose tissue function [53,54]. Moreover, through a regulatory feedback loop, they influence *Pparγ* gene expression and thereby modulate the later stages of adipogenic progression [55,56,57]. Given the central role of *Pparγ* in adipocyte maturation, we hypothesize that its downregulation lies at the core of the DHA-induced anti-adipogenic effect observed in immature adipocytes—an effect that, at first glance, appears to challenge the widely held assumption that omega-3 fatty acids act as *Pparγ* agonists.

DHA is well-known for its strong antioxidant and anti-inflammatory properties [4,7,18,58]. However, it is also a well-established fact that a moderate level of inflammation is an absolutely necessary trigger for adipogenesis. During early adipogenic induction (in vitro, via a hormonal cocktail containing insulin, dexamethasone, indomethacin, and IBMX), both cAMP/PKA and glucocorticoid-dependent signaling pathways are activated. These pathways rapidly induce the expression and phosphorylation of early transcription factors C/EBPβ and C/EBPδ. Their stabilization and activity are strongly dependent on reactive oxygen species (ROS) signaling, which plays a critical role in initiating the transcription of *Pparγ*, and subsequently C/EBPα, both of which are master regulators of the transition from preadipocyte to mature adipocyte [59,60].

DHA supplementation at the onset of adipogenic differentiation leads to its intensive incorporation into both the plasma and mitochondrial membranes. This enhances their fluidity, remodeling their lipid rafts, alters the size and composition of the microdomains, thereby modulating the activity of key signaling molecules, such as G-protein-coupled receptors (GPCRs) [61]. Scientific studies reveal that DHA decreases the activity of NADPH oxidase (NOX4), thereby lowering superoxide levels and the overall production of ROS [62,63,64]. The suppressed oxidative stress could limit the coactivation of stress kinases at the CRE/AP-1/κB response elements, thereby may reduce the early induction of C/EBPβ and C/EBPδ [59,65,66].

At the same time, a fraction of intracellular DHA is oxidized into electrophilic lipid-derived metabolites (such as 4-hydroxy-2-hexenal), which can modify Keap1 and thereby promote the stabilization and nuclear translocation of Nrf2. This, in turn, triggers the ARE-dependent antioxidant genes, including *SOD*, GPx, and catalase/HO-1 [58,67,68]. This literature based mechanism supports the notion that DHA can enhance cellular redox homeostasis independently of the external adipogenic stimuli [68].

Indirect effects via AMPK activation have also been described including reduction in inflammatory response and the attenuation of transcriptional activation required for the expression of C/EBPβ, C/EBPδ, and *Pparγ* [66,68]. Although these pathways were not directly assessed in the present study, they provide a valuable context for interpreting the observed decreased expression of *Pparγ* and its downstream adipogenic targets, such as *Fabp4*, *Gpr120*, and *Adipoq*, observed in DHA-treated adipocytes during adipogenesis.

In addition, AMPK signaling also plays a crucial role in controlling the activity of SREBP-1c—the second major transcriptional regulator of adipogenesis by preventing its nuclear import and reducing the transcription of its lipogenic targets such as *Fasn* and *Acaca* [69]. *Acaca* catalyzes the conversion of acetyl-CoA to malonyl-CoA, a critical precursor for de novo lipogenesis and a known inhibitor of *Cpt1*. AMPK-mediated phosphorylation of *Acaca* blocks malonyl-CoA synthesis, thereby shifting metabolism to favor mitochondrial β-oxidation over lipogenesis. Consistent with this mechanism, we established upregulation of *Cpt1* and downregulation of *Fasn* gene expression in the DM-DHA group. Our data also indicated that the gene expression of *Srebf1* was significantly decreased in DM_DHA group, while it remained unchanged in the MM_DHA group. Taken together, the above-described mechanisms provide reasonable support of concept that DHAexert stage-dependent regulatory effects in adipocytes.

The outcomes established in DM_DHA 3T3-L1 cells suggest potential benefits for weight loss in vivo. However, we noted not only a significant decrease in lipid accumulation and lipolysis rate but also an approximately 44% reduction in glucose uptake by mature adipocytes—a logical consequence of the suppressed expression levels of the *Pparγ*, *Gpr120*, and *Slc2a4* genes. Considering the high-carbohydrate feeding mimicked in our experiment, such metabolic interference at the organism level could potentially contribute to glucotoxicity, insulin resistance, and type 2 diabetes. Outdated, the pharmacological suppression of PPARγ has been used to manage obesity-associated complications. This strategy is now reevaluated in favor of approaches that enhance the buffering capacity of adipose tissue, particularly in individuals with pre-existing obesity or a high metabolic risk profile [70,71,72].

Interestingly, the same effects of DHA that may pose risks under conditions of metabolic overload appear to be highly beneficial in a different pathological context. In cancers, where a sustained energy supply and lipogenic signaling are essential for tumor growth and survival, DHA-induced restriction of energy influx, together with the downregulation of *Pparγ*, *Fabp4*, *Fasn*, *Gpr120*, and *Slc2a4*, disrupts key pro-tumorigenic pathways. These findings align with previous reports, which outline DHA as a nutritional additive with the potential to reduce SREBP1/FASN signaling, thereby further inducing tumor cell death programs [73,74,75].

PPARγ initiates early adipogenesis and later becomes a key regulator of adipose tissue homeostasis in mature adipocytes. Its downregulation at this stage leads to maladaptive hypertrophy, mitochondrial dysfunction, and inflammation, and may even induce apoptosis [51,76].

In our study, DHA supplementation in mature adipocytes exerted no effect on the mRNA expression of *Pparγ*, *Fabp4*, *Fasn*, *Adipoq*, or *Lep*, nor did it affect adiponectin protein levels. The *Gpr120* and *Slc2a4* (GLUT4) gene expression was downregulated, but this was accompanied by only an 8% reduction in glucose uptake—a negligible effect compared to the 44% decrease observed in immature (DM_DHA) adipocytes. Notably, the 30% decrease in intracellular lipid accumulation (compared to DC) was coincident with a 34% increase in lipolysis, suggesting that the DHA provoked triglyceride depletion in mature adipocytes primarily reflects enhanced lipid mobilization rather than a shift toward increased β-oxidation.

From a systemic perspective, the DHA-enhanced lipolysis in mature adipocytes—widely documented in vitro [28,77]—may elevate circulating NEFA levels, potentially promoting ectopic lipid deposition and lipotoxicity in peripheral tissues such as the liver and skeletal muscle. This risk, however, may be mitigated if hepatic β-oxidation is simultaneously upregulated—a response frequently attributed to DHA via PPARα-mediated induction of CPT1 and ACOX1. Further investigations in vitro are required to determine whether, and at what dosage, DHA administration under conditions of pre-existing obesity and chronic energy surplus can exert systemic effects potent enough to counteract the potential metabolic risks associated with increased NEFA release.

This study primarily examines the transcriptional response of key adipogenic markers linked to *Pparγ* regulation. A limitation of the current work is the absence of protein-level analyses and advanced lipid assessment methods, which would provide deeper mechanistic insight. Future studies are needed to achieve a more comprehensive characterization of DHA’s metabolic effects at different stages of adipogenesis.

## 4. Materials and Methods

### 4.1. Materials and Chemical Reagents

The current study utilized 3T3-L1 mouse embryonic fibroblasts (ATCC CRL-3242) from the American Type Culture Collection (ATCC, Washington, DC, USA). The reagents used in the current investigation were Dulbecco’s Modified Eagle’s Medium (DMEM) with high glucose content (4500 mg/L); fetal bovine serum (FBS); L-glutamine; antibiotic solution (Penicillin G, Streptomycin, Amphotericin B); indomethacin; dexamethasone; docosahexaenoic acid (C22:6) (DHA); palmitic acid (C10:0) (PA); phosphate-buffered saline (PBS); 100% ethanol, isopropanol; Oil Red O powder; 3-(4,5-dimethyl-2-thiazolyl)-2,5-diphenyl-2H-tetrazolium bromide (MTT) powder; trypsin; dimethyl sulfoxide (DMSO); Trypan blue dye; glucose analysis kit for cell culture supernatants (MAK013); lipolysis analysis kit for cell culture supernatants (MAK313); the enzyme-linked immunosorbent assay (ELISA) (RAB1115)—all suitable for cell cultures and purchased from Sigma-Aldrich, Chemie, GmbH (Merk KGaA, Darmstadt, Germany). Insulin (cell application, San Diego, CA, USA) and 3-isobutyl-1-methylxanthine IBMX (Cayman Chemical, Ann Arbor, MI, USA) were also used. The plates and pipettes used were sterile and single-use, produced by Corning Incorporated, Costar, Corning, NY, USA. For gene expression analyses, we used the RNeasy Mini Lipid Tissue Kit (QIAGEN GmbH, Hilden, Germany), the RevertAid First Strand cDNA Synthesis Kit (Thermo Fisher Scientific, Waltham, MA, USA), and the Luminaris HiGreen qPCR Master Mix (Thermo Fisher Scientific).

### 4.2. Composition of the Culture Media

The 3T3-L1 cell line was cultured in media with three different compositions:

Basal medium (BM)—composed of DMEM, 2% L-glutamine, 10% (*v*/*v*) FBS, and 1% antibiotic solution; Differentiating medium (ADM)—consisting of DMEM, 2% L-glutamine, 10% (*v*/*v*) FBS, 0.1 mM IBMX, 0.05 mM indomethacin, 1 µM dexamethasone, 10 µg/mL insulin, and 1% antibiotic solution; Adipocyte maintenance medium (AMM)—DMEM, 2% L-glutamine, 10% (*v*/*v*) FBS, 10 µg/mL insulin, and 1% antibiotic solution.

### 4.3. Fatty Acid Dissolving Procedure

In the current experiment, ethanol-dissolved fatty acids were utilized as an alternative to the more commonly used bovine serum albumin conjugates. This approach ensures direct exposure to the free fatty acid fraction and helps prevent potential alterations in adipogenic and lipolytic processes, as albumin has been shown to influence adipogenic signaling [78].

PA or DHA were first dissolved in 100% ethanol and validated in cell cultures. The prepared stock solutions were stored at −20 °C until use. Immediately before treatment, the PA or DHA stock solution was further diluted with the culture medium in a controlled, step-by-step manner to achieve the desired final concentration of 60 µM. This stepwise protocol resulted in a final ethanol concentration of 0.05% (*v*/*v*) within the culture media. The concentrations of PA, DHA, and ethanol implemented in this study were selected based on cell viability outcomes from assays previously described by Grigorova et al. [43]. Here, we evaluated PA markedly reduced 3T3-L1 cell viability at 80 μM, while DHA caused only a modest, non-significant decrease at 80 μM and a significant reduction at 100 μM. Since the present experiment includes both fatty acids, 60 μM was identified as the highest concentration that does not compromise 3T3-L1 viability and was therefore selected for all treatments.

### 4.4. Pre-Experimental Procedure: Effect of 0.05% (v/v) Ethanol on Adipogenesis and Lipolysis in Differentiating and Mature Adipocytes

To evaluate any potential effects of lipolysis and intracellular lipid accumulation resulting from extended adipocyte exposure to 0.05% (*v*/*v*) ethanol, a preliminary experiment was conducted following the protocol outlined in Table 1. 3T3-L1 preadipocytes were initially cultured with BM in T75 flasks at 37 °C, a humidified atmosphere of 95% air and 5% CO_2_. At 80% confluence, the cells were seeded into 24-well plates at a density of 10^4^ cells per well and cultured until they reached 100% confluence. Subsequently, the preadipocytes underwent a 24 h growth arrest before being subjected to adipogenic differentiation, which involved an initial 2-day period in ADM followed by a 16-day incubation in AMM. A 0.05% (*v*/*v*) ethanol was added to the culture medium for nine days: from day 1 to day 9 in the first experimental group (DM-eth) and from day 10 to day 18 in the second group (MM-eth). Outcomes were quantified relative to a non-treated, differentiated control group (DC), expressed as a percentage. At the end of the experimental period (day 18), assessments were made of the degree of intracellular lipid accumulation and the rate of lipolysis.

### 4.5. Experimental Design

3T3-L1 preadipocytes were propagated in BM and then seeded in 12- and 24-well plates at a concentration of 10^4^ cells/well. The experimental layout is presented in Table 2. The cells were grown and seeded under the same conditions, as described above. Upon reaching 100% confluence, the cells were subjected to a 24 h growth arrest and were divided into two main groups: undifferentiated (negative controls) (NC) and differentiated cells (DC). The NC was maintained in a BM throughout the experiment. The baseline levels of adipogenesis, lipolysis, and glucose uptake, elicited solely by the availability of high glucose, were evaluated in this group. These baseline values were subsequently subtracted from the data obtained from all other experimental groups. The remaining preadipocytes underwent a 48 h induction in ADM and were further differentiated for 16 days in AMM. To compare the impact of DHA on the 3T3-L1 adipogenesis at different stages of cell maturity, 60 µM DHA was introduced into the culture medium either at the onset of induction (day 1) or to fully mature adipocytes (day 10), spanning nine days (indicated as DM_DHA and MM_DHA, respectively). Isoenergetic controls (DM_PA and MM_PA) were supplemented with palmitic acid (PA) under identical conditions.

For each group, four parallel experiments were conducted concurrently: (1) Oil Red O staining (12-well plates); (2) MTT assay (24-well plates); (3) Trypan blue exclusion assay (24-well plates); and (4) cell culture supernatants analysis for quantification of glucose, glycerol and adiponectin levels, together with mRNA isolation for RT-qPCR (12-well plates).

The culture media were changed every 48 h throughout the experiment, with sampling conducted at the end of the trial.

### 4.6. Cell Viability Assays

Cell viability was assessed using an MTT assay, as described by Yang et al. [79]. This colorimetric assay measures the percentage of viable cells by assessing the reduction of MTT through NAD(P)H-dependent cellular oxidoreductase enzymes, which results in the formation of an insoluble, purple-colored formazan product. Its absorbance was measured at 570 nm (with a reference wavelength of 630 nm) [80] and expressed as a percentage of NC [81].

Cell viability (%) = [OD570 (sample)/OD570 (NC)] × 100(1)

To affirm the results obtained upon the MTT test, a Trypan blue exclusion assay was conducted. The technique was based on a modified protocol described by Danesi et al. [82]. The percentage of viable cells was calculated with the formula:

% Viable cells = [1 − (Number of blue-stained cells/Total cell count)] × 100(2)

Subsequently, cell viability was quantified relative to NC, and results were expressed as a percentage thereof.

### 4.7. Oil Red O Staining and Evaluation of Intracellular Lipid Accumulation

The intracellular accumulation of lipids in all cell groups was Visualized and quantified using Oil Red O staining followed by isopropanol extraction. The staining procedure was described previously by Yang et al. [79]. Intracellular lipid deposition was captured using a Leica inverted microscope for cell and tissue cultures, equipped with a 5-megapixel camera (DMi1). The final step involved the dye extraction with isopropanol and the spectrophotometric determination of absorbance at a wavelength of 490 nm using a Synergy^TM^ LX Multi-Mode Microplate Reader (BioTek Instruments, Inc., Winooski, VT, USA). All data obtained were expressed relative to the DC after subtracting the values of the NC.

### 4.8. Glucose Uptake

The extracellular glucose concentration (EG) was determined using a cell supernatant analysis kit (MAK013). Samples and standards were each analyzed in duplicate according to the manufacturer’s instructions. The absorbance of the colorimetric product was measured at 530 nm using a Synergy^TM^ LX Multi-Mode Microplate Reader (BioTek Instruments, Inc., USA). Glucose concentration was estimated from the standard curve after correcting for the blank. Cell-free culture media corresponding to each group were maintained under identical conditions and analyzed concurrently with the experimental supernatants. These initial glucose concentrations (IG) were used in the calculation of glucose uptake in mmol/L, employing the evaluation equation described by Diaz et al. [83]:

Glucose uptake (mmol/L) = IG − EG(3)

Glucose uptake percentages were evaluated by subtracting the basal glucose uptake in NC (BG_NC_) from the glucose uptake in each induced group (SG_i_). Subsequently, the results were normalized to DC (SG_DC_). The formula used for glucose uptake (%) was as follows:

Glucose uptake (%) = (EG_i_ − BG_NC_)/EG_AIM_ × 100 (4)

### 4.9. Lipolysis Rate

The MAK313 analysis kit was employed to estimate glycerol concentration in cell culture supernatants. Each sample (10 µL) was measured in duplicate according to the manufacturer’s protocol. Optical density (OD) was measured at 570 nm with correction at 630 nm, using a Synergy^TM^ LX Multi-Mode Microplate Reader (BioTek Instruments, Inc., USA). Glycerol concentration was calculated in µg/mL based on the OD of the standard curve. The percentage of lipolysis was then determined relative to the AIM after adjusting for the basal lipolysis established in the NC.

### 4.10. Gene Expression Analysis

mRNA was extracted using the RNeasy Mini Kit for Lipid Tissue and strictly following the manufacturer’s protocol. RNA quantity and quality were measured spectrophotometrically at absorbances of 260 and 280 nm using a Synergy^TM^ LX Multi-Mode Microplate Reader equipped with a Take3 Microvolume Plate (BioTek Instruments, Inc., Winooski, VT, USA). Following uniform sample concentration, reverse transcription was performed using the RevertAid First Strand cDNA Synthesis Kit, and the resulting cDNAs were stored at −20 °C.

Primer pairs either for the housekeeping genes (hydroxymethylbilane synthase (*Hmbs*), beta-actin (*Actb*), ribosomal protein, large, P0 (*36b4*), hypoxanthine guanine phosphoribosyl transferase (*Hprt*) and 18S ribosomal RNA (*18S*)) and target genes (*Pparγ*, *Srebf1*, *Gpr120*, *Pnpla2*, *Lipe*, *Fabp4*, *Fasn*, *Slc2a4*, *Cpt1*, *Lep*, and *Adipoq*) were designed using Primer 3 (version 4.1.0) (https://primer3.ut.ee/) and National Center for Biotechnology Information resources (NCBI) (https://www.ncbi.nlm.nih.gov), aligning with the criteria described in the Luminaris HiGreen qPCR Master Mix (Thermo Fisher Scientific) instructions for use. The *18S* primer sequence was reported initially by Arnhold et al. [84]. Primer specificity was subsequently confirmed using Primer-BLAST (Basic Local Alignment Search Tool—NCBI) (https://www.ncbi.nlm.nih.gov/tools/primer-blast, accessed on 21 October 2025), Primer 3plus (version 3.3.0) (https://www.primer3plus.com/index.html, accessed on 21 October 2025), and SerialCloner (version 2.6.1). Prognostic product characteristics, including melting temperature and curves, were analyzed by uMelt Quartz (version: 3.6.2 “Quartz”/5 November 2020) (https://www.dna-utah.org/umelt/quartz/um3.php, accessed on 21 October 2025). All software tools were assessed on 12 May 2022 and 21 October 2024. The specific primer sequences used in this experiment are described in Table 3. 

A two-step protocol of real-time PCR analysis was performed to assess relative gene expression using a thermocycler RT-qPCR System (PikoReal, Thermo Fisher Scientific, Waltham, MA, USA). Following the temperature program recommended by the master mix manufacturer, RT-qPCR reactions were conducted in duplicate. PCR product validity was affirmed through an evaluation of the melting curves. A standard curve-based efficiency of each gene expression was also computed. RT-qPCR efficiency ranged from 97% to 102%.

The stability of the candidate reference genes was evaluated using four validation tools: NormFinder (Excel add-in, MS Office 2016) [85], the Excel-based geNorm macro (geNorm v3.5, Windows/VBA, Ghent, Belgium: Center for Medical Genetics, Ghent University, https://genorm.cmgg.be/) [86], BestKeeper v1, an Excel-based tool (Munich, Germany: Technical University of Munich, Institute of Physiology), https://www.gene-quantification.de/bestkeeper.html (accessed on 21 October 2025) [87], and RefFinder https://www.ciidirsinaloa.com.mx/RefFinder-master/ (accessed on 21 October 2025) [88]. All accessed during the period 1–8 October 2025. The results indicated that the combination of Actb, 18S, and 36b4 constituted the most stable reference genes. Therefore, gene expression levels were relatively quantified using the modified 2^−ΔΔCt^ method, with normalization applied using the geometric mean of the selected reference genes [89,90].

### 4.11. ELISA Adiponectin Assessment

We determined the adiponectin concentrations in the cell culture supernatants using a sandwich ELISA procedure, which was carried out according to the manufacturer’s instructions. Adiponectin levels were quantified in picograms per milliliter (pg/mL) according to the optical density (OD) readings from the standard curve. These readings were obtained using a Synergy^TM^ LX Multi-Mode Microplate Reader. The relative adiponectin expression levels were then calculated as a percentage of the DC.

### 4.12. Statistical Analysis

All data were analyzed using the software system—Statistica version 10 (StatSoft, Inc., Tulsa, OK, USA (2011)). A standard descriptive data processing method was employed, and the final results are presented as the mean value and standard error of the mean (SEM). The significance of the differences between the studied groups was evaluated using the nonparametric Mann–Whitney test. *p*-values less than 0.05 were considered statistically significant.

## 5. Conclusions

Treatment with 60 µM DHA under high-carbohydrate conditions (a classical in vitro model of adipogenesis) reduced the lipid-buffering capacity of both differentiating and mature 3T3-L1 adipocytes without affecting cell viability. It could appear beneficial by limiting lipid storage; however, in the context of persistent energy surplus, such suppression of adipogenesis could compromise the protective role of adipose tissue and increase the risk of metabolic complications. Therefore, DHA’s modulation of adipogenesis at high physiological concentrations should be approached with caution to avoid unintended adverse effects.

## Figures and Tables

**Table 1:** Experimental design overview of the preliminary experiment.

Stages/Groups	DC	DM-eth	MM-eth
Day 1–2 (48 h)	INDUCTION in ADM	INDUCTION in ADM + 0.05% (*v*/*v*) ethanol	INDUCTION in ADM
Day 3–9	Preadipocyte maturation in AMM	Maturation of preadipocytes in AMM + 0.05% (*v*/*v*) ethanol	Maturation of preadipocytes in AMM
Day 10–18	Mature adipocyte growth in AMM	Growth of mature adipocytes in AMM	Growth of mature adipocytes in AMM + 0.05% (*v*/*v*) ethanol

**Table 2:** Experimental design.

Stages/Groups	NC	DC	DM_PA/DM_DHA	MM_PA/MM_DHA
Day 0 (24 h)	Growth arrest at 100% confluence			
Day 1–2 (48 h)	Preadipocyte culture in BM	INDUCTION in ADM	INDUCTION in ADM + 60 µM DHA	INDUCTION in ADM
Day 3–9		Preadipocyte maturation in AMM	Preadipocyte maturation in AMM + 60 µM PA or DHA	Preadipocyte maturation in AMM
Day 10–18		Mature adipocytes cultured in AMM	Mature adipocytes cultured in AMM	Mature adipocytes cultured in AMM + 60 µM PA or DHA

**Table 3:** Gene-specific primer sequences, gene accession numbers, and corresponding RT-qPCR product sizes.

Abbreviation	Full Name	Forward Primer	Reverse Primer	Product Size (bp)
*Pparγ* NM_001127330.2	Peroxisome proliferator-activated receptor gamma	AGGGCGATCTTGACAGGAAA	CGAAACTGGCACCCTTGAAA	164
*Srebf1* NM_011480.4	Sterol regulatory element binding transcription factor 1	TTGACACGTTTCTTCCTGAGC	CAGTTCAACGCTCGCTCTAG	239
*Gpr120* NM_181748.2	G protein-coupled receptor 120	CCAACCGCATAGGAGAAATC	CAAGCTCAGCGTAAGCCTCT	140
*Pnpla2* NM_001163689.1	Patatin-like phospholipase domain containing 2	CCTTCACCATCCGCTTGTTG	CCCAGTGAGAGGTTGTTTCG	250
*Lipe* NM_010719.5	Lipase, hormone-sensitive	ACGAGCCCTACCTCAAGAAC	GCTCTCCAGTTGAACCAAGC	165
*Lep* NM_146146.3	Leptin receptor, transcript variant 1	GAGCCCCAAACAATGCCTC	TGTCCCAGTTTACACCTAGCT	231
*Adipoq* NM_028320.4	Adiponectin receptor 1	TCCCGTATGATGTGCTTCCT	AGCACAAAACCAAGCAGATGT	157
*CPT1* NM_153679.2	Carnitine palmitoyltransferase 1	GTGTACTTCCAACTACGTCAGC	GCGATACAGGAGCAGGGTAT	169
*FABP4* NM_024406.4	Fatty acid-binding protein 4	AACTGGGCGTGGAATTCGAT	CCACCAGCTTGTCACCATCT	150
*Fasn* NM_007988.3	Fatty acid synthase	CTGAAGCCGAACACCTCTGT	GGGAATGTTACACCTTGCTCCT	218
*Slc2a4* NM_009204.2	Solute carrier family 2 member 4	CGTTGGTCTCGGTGCTCTTA	AGCTCTGCCACAATGAACCA	220
*Hmbs* NM_001110251.1	Hydroxymethylbilane synthase	CCTGAAGGATGTGCCTACCA	CCACTCGAATCACCCTCATCT	175
*36b4* NM_007475.5	Ribosomal protein, large, P0	TTATAACCCTGAAGTGCTCGAC	CGCTTGTACCCATTGATGATG	147
*Hprt* NM_013556.2	Hypoxanthine guanine phosphoribosyl transferase	ACAGGCCAGACTTTGTTGGA	ACTTGCGCTCATCTTAGGCT	150
*18S* NR_046271.1	18S ribosomal RNA	ATGCGGCGGCGTTATTCC	GCTATCAATCTGTCAATCCTGTCC	204
*Actb* NM_007393.5	β-actin	CCTCTATGCCAACACAGTGC	GTACTCCTGCTTGCTGATCC	211

## Data Availability

The original contributions presented in this study are included in the article. Further inquiries can be directed to the corresponding author.

## Abbreviations

The following abbreviations are used in this manuscript:

3T3-L1	Mouse embryonic fibroblast cell line used as an adipogenesis model
*18S*	18S ribosomal RNA
*36b4*	Ribosomal protein, large, P0
*Acaca*	Acetyl-CoA carboxylase alpha
*Adipoq*	Adiponectin
AMPK	AMP-activated protein kinase
*Actb*	Beta-actin
ACOX1	Acyl-CoA oxidase 1
ADM	Adipocyte differentiating medium (untreated control)
ADM-Eth	Differentiating cells treated with 0.05% (*v*/*v*) ethanol (days 1–9)
AMM	Adipocyte maintenance medium
AMM-Eth	Mature adipocytes treated with 0.05% (*v*/*v*) ethanol (days 10–18)
ATGL	Adipose triglyceride lipase (encoded by Pnpla2)
BM	Basal medium
C/EBPα	CCAAT/enhancer-binding proteins alpha
C/EBPβ	CCAAT/enhancer-binding proteins beta
C/EBPδ	CCAAT/enhancer-binding proteins delta
*Cpt1*	Carnitine palmitoyltransferase 1
CREB	cAMP response element-binding protein
CRTC2	CREB-regulated transcription coactivator 2
cAMP/PKA	Cyclic adenosine monophosphate/protein kinase A signaling pathway
cDNA	Complementary deoxyribonucleic acid
Ct	Cycle threshold
DC	Differentiated control
DHA	Docosahexaenoic acid
DM_PA	Differentiating cells treated with 60 µM palmitic acid (days 1–9)
DM_DHA	Differentiating cells treated with 60 µM docosahexaenoic acid (days 1–9)
DMEM	Dulbecco’s Modified Eagle’s Medium
DMSO	Dimethyl sulfoxide
ΔΔCt	delta delta Ct
EG	Extracellular glucose concentration
ELISA	Enzyme-linked immunosorbent assay
*Fabp4*	Fatty acid-binding protein 4
*Fasn*	Fatty acid synthase
FBS	Fetal bovine serum
*Gpr120*	G protein-coupled receptor 120 (gene)
GPR120	G-protein-coupled receptor 120 (protein)
GPCRs	G-protein-coupled receptors
GPx	Glutathione peroxidase
*Hmbs*	Hydroxymethylbilane synthase
*Hprt*	Hypoxanthine-guanine phosphoribosyl transferase
HSL	Hormone-sensitive lipase (encoded by Lipe)
IBMX	3-Isobutyl-1-methylxanthine
IG	Initial glucose concentration
*Lep*	Leptin receptor, transcript variant 1
*Lipe*	Lipase, hormone-sensitive
MTT	3-(4,5-dimethylthiazol-2-yl)-2,5-diphenyltetrazolium bromide
MM_PA	Mature adipocytes treated with 60 µM palmitic acid (days 10–18)
MM_DHA	Mature adipocytes treated with 60 µM docosahexaenoic acid (days 10–18)
mTORC1	Mammalian target of rapamycin complex 1
NADPH	Nicotinamide adenine dinucleotide phosphate
NC	Non-differentiated control
NEFA	Non-esterified fatty acids
NOX4	NADPH oxidase 4
Nrf2	Nuclear factor erythroid 2-related factor 2
PA	Palmitic acid
PBS	Phosphate-buffered saline
PKA	Protein kinase A
*Pnpla2*	Patatin-like phospholipase domain-containing 2
PPARγ	Peroxisome proliferator-activated receptor gamma (protein)
*Pparγ*	Peroxisome proliferator-activated receptor gamma (gene)
RT-qPCR	Reverse transcription quantitative polymerase chain reaction
PUFAs	Polyunsaturated fatty acids
RNA	Ribonucleic acid
ROS	Reactive oxygen species
RT-PCR	Reverse transcription polymerase chain reaction
SEM	Standard error of the mean
*Slc2a4*	Solute carrier family 2 member 4 (encoding glucose transporter 4, GLUT4)
SOD	Superoxide dismutase
*Srebf1*	Sterol regulatory element binding transcription factor 1
SREBP-1c	Sterol regulatory element-binding protein 1c
